# A density functional theory study of the molecular structure, reactivity, and spectroscopic properties of 2-(2-mercaptophenyl)-1-azaazulene tautomers and rotamers

**DOI:** 10.1038/s41598-023-42450-1

**Published:** 2023-09-20

**Authors:** Safinaz H. El-Demerdash, Shimaa Abdel Halim, Ahmed M. El-Nahas, Asmaa B. El-Meligy

**Affiliations:** 1 Chemistry Department, Faculty of Science, Menoufia University, Shebin El-Kom, 32512 Egypt; 2 Chemistry Department, Faculty of Education, Ain Shams University, Cairo, Egypt

**Keywords:** Computational science, Chemistry

## Abstract

Five stable tautomer and rotamers of the 2-(2-Mercaptophenyl)-1-azaazulene (thiol, thione, R1, R2, and R3) molecules were studied using density functional theory (DFT). The geometries of the studied tautomer and rotamers were fully optimized at the B3LYP/6-31G(d,p) level. Thermodynamic calculations were performed at M06-2X/6-311G++(2d,2p) and ωB97XD/6-311G++(2d,2p) in the gas phase and ethanol solution conditions modeled by the solvation model based on density (SMD). The kinetic constant of tautomer and rotamers conversion was calculated in the temperature range 270–320 K using variational transition state theory (VTST) accompanied by one-dimensional wigner tunneling correction. Energy refinement at CCSD(T)/6–311++G(2d,2p) in the gas phase has been calculated. All the studied DFT methods qualitatively give similar tautomer stability orders in the gas phase. The ethanol solvent causes some reordering of the relative stability of 2-(2-Mercaptophenyl)-1-azaazulene conformers. The transition states for the 2-(2-Mercaptophenyl)-1-azaazulene tautomerization and rotamerization processes were also determined. The reactivity, electric dipole moment, and spectroscopic properties of the studied tautomer and rotamers were computed. The hyper-Rayleigh scattering (*β*_*HRS*_), and depolarization ratio (DR) exhibited promising optical properties when nonlinear optical properties were calculated.

## Introduction

Tautomerism provides knowledge of the variety of forms in which organic molecules can exist. Many biologically active compounds, including pyrazolone derivatives, take part in tautomeric transformation^1^. In numerous fields of chemistry, intramolecular proton transfer (IPT) is crucial^2^. Its mechanism takes place in both ground and excited states^3^. Compounds that exhibit intramolecular proton transfer can be used as laser dyes, fluorescent probes, high-energy radiation detectors, memory storage devices, and polymer protectors^4^. Hence, molecules that have intramolecular proton transfer have received great interest from an experimental and theoretical perspective^5,6^. Numerous topics in organic chemistry and biochemistry can be studied more effectively by understanding the nature of tautomeric equilibrium^7^.

Thione–thiol tautomerization is a remarkable subject that has attracted a lot of study. The thione tautomer is mostly detected in the gas phase under standard experimental conditions. Thiol tautomers of thione compounds, which can be stabilized in inert matrices at lower temperatures, can be subjected to UV light. These compounds include (1H)-pyridinethione^8^, 4(3H)-pyrimidinethione and 3(2H)-pyridazinethione^9^, 2(1H)-quinolinethione^10^, methimazole^11^, 2-thiobenzimidazole^12^, thiourea^13^, and thioacetamide^14^. Thiophenol is a notable exception that only exists as a thiol tautomer^15^. Organosulfur compounds containing thiocarbonyl groups have attracted interest because of their diverse chemistry^16^, various biological applications^17,18^, and the ability of the C = S bond to oxidize quickly to form the corresponding thiols and disulfides^19^.

**Figure 1:**
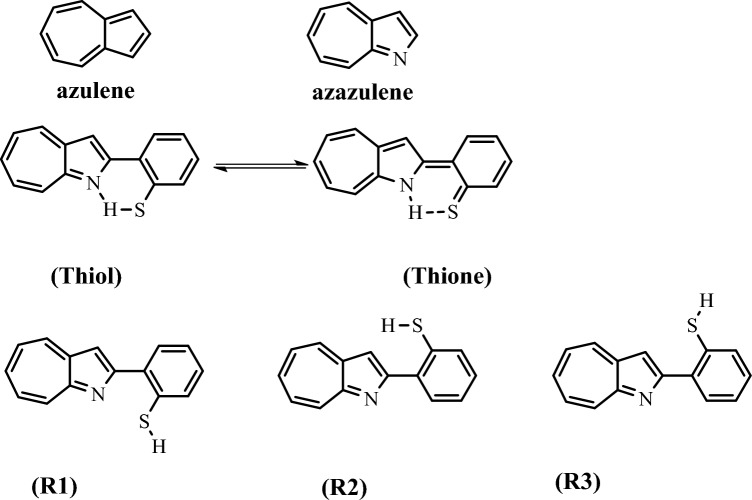
The tautomeric and rotameric structures of 2-(2-Mercaptophenyl)-1-azaazulene.

Azaazulenes have attracted a lot of attention because of their chemical, physical, and biological activities^20^. Azaazulene is an aromatic heterocyclic compound. It stands for a significant class of compounds with systems of π- and n-electrons. Oda et al.^21^ investigated 2-(2-Hydroxyphenyl)-1-azaazulene's production, molecular structure, and characteristics. In addition, the structures, energetics, and spectra of 2-(2-Hydroxyphenyl)-1-azaazulene tautomers have been examined using B3LYP, M06-2X, B97XD, and CCSD(T) with various basis sets^22^. The biological potential of its counterpart, 2-(2-Mercaptophenyl)-1-azaazulene, is anticipated. In order to fully understand the researched compound's expected distinctive biological properties, its electronic structure must also be investigated, along with its electronic and optical spectra. The 2-(2-Mercaptophenyl)-1-azaazulene structure is classified as an organic semiconductor of a small molecule and is designed as a π-conjugated nanostructure. It also has a significant extinction coefficient, good light gain, and a delocalization of electrons. These materials are suitable for optoelectronic devices as a result of their highly appealing electrical and optical properties^23^. There haven’t been any theoretical or experimental studies on 2-(2-Mercaptophenyl)-1-azaazulene. So, we will study the thiol-thione tautomerization of 2-(2-Mercaptophenyl)-1-azaazulene (Fig. 1) in the gas phase and ethanol.

The standard methods for studying tautomeric transformations in solutions and in the crystalline state include X-ray crystallography, nuclear magnetic resonance spectrometry, ultraviolet radiation, infrared spectrophotometry, and quantum chemistry^24,25^. Using DFT and TD-DFT, quantum-chemical calculations can estimate minimal computational needs and provide excellent findings from experiments^26,27^. As a result, they are particularly interesting for researching tautomeric compounds^26,27^. However, the results always remain confined to the level of the theory and basis sets that were employed^28,29^.

In the current study, we use the B3LYP functional in the DFT approach to study 2-(2-Mercaptophenyl)-1-azaazulene’s molecular structure, thermodynamic and kinetic stabilities for the first time. Additionally, equilibrium constants, reactivity, and spectroscopic properties of the different tautomeric structures of 2-(2-Mercaptophenyl)-1-azaazulene will be studied using different quantum chemical methods. The electrical and optical properties of these tautomers are also shown by NLO analysis, which also explains charge transport inside them.

## Computational details

The commonly used B3LYP method has provided the correct molecular geometries, activation energies, and energy differences between pairs of tautomers, similar to MP2-level predictions^30^. It has been concluded that the small absolute errors and the cost-effectiveness of the computations make the B3LYP method a good choice for these systems^30^. Our computations were carried out using the gradient-corrected hybrid density functional B3LYP DFT method^31^. For each structure, a full geometry optimization without symmetry constraints was performed using this functional along with the 6-31G(d,p) basis set^32^ as implemented by the Gaussian 09 package^33^. All geometries were visualized either using GaussView 5.0.9^34^ or ChemCraft 1.6^35^ programs.

The presence of one imaginary frequency that the ChemCraft program evaluated corroborated the presence of TSs^35^. Real frequencies are shown by Minima. Using the 6–311++G(2d,2p) basis set at the B3LYP/6-31G(d,p) optimized structures, energies were adjusted at the meta-hybrid generalized gradient approximation (M06-2X)^36,37^ and long-range corrected hybrid functional of Becke's 97 that incorporates dispersion correction (ωB97XD)^38,39^ functionals. The CCSD(T)^40^ computations are a potent tool for precise estimation of reaction and activation energies for species when a single-reference wavefunction is a good approximation. As a result, we used the B3LYP/6–31+G(d,p) geometries to do single point energy calculations at CCSD(T)/6–311 ++G(2d,2p). The findings show that, in comparison to the more precise (CCSD(T)/6–311++G(2d,2p) level, ωB97XD/6–311++G(2d,2p) performs marginally better than M06-2X/6–311++G(2d,2p). Therefore, for discussion of energetics, the level ωB97XD/6–311+ +G(2d,2p) is used unless otherwise stated.

The solvation effect has been modeled in ethanol using the solvation model based on density (SMD)^41^ at the B3LYP/6–31+G(d,p) level for all the tautomers and rotamers. We estimated solvation energies in ethanol as single point energy calculations on geometries optimized in ethanol at B3LYP using M06-2X, B3LYP, and ωB97XD functionals with 6–311++G(2d,2p) basis sets.

On the basis of the statistical thermodynamics principle^42^, the entropy (*S*) and enthalpy (*H*) of the tautomers in all environments at standard conditions of *T* = 298.15 K and *P* = 1 atm have been obtained. Gibbs free energy values, which were calculated at the same levels of theory, were used for the evaluation of the tautomeric equilibrium constants. For the tautomerization and rotamerization transformation processes, rate coefficients were calculated in the temperature range 270–320 K (*k*_*uni*_, in s^−1^) using the variational transition state theory (CVT)^43^ that includes generalized transition state rates $k_{{}}^{GT}$ with wigner tunneling correction ($\chi (T)$)^44^ according to (1–3) equations.

1 $$ k^{GT} (T) = \sigma \tfrac{{K_{b} T}}{h}\tfrac{{Q^{TS} (T,s)}}{{N_{A} Q^{R} (T)}}e^{{{\raise0.5ex\hbox{$\scriptstyle { - V^{\# } (S)}$} \kern-0.1em/\kern-0.15em \lower0.25ex\hbox{$\scriptstyle {K_{b} T}$}}}} $$

2 $$ k_{{}}^{CVT} (T) = \min K^{GT} (T,s) $$

3 $$ \chi (T) = 1 + \frac{1}{24}(\frac{h\nu }{{K_{b} T}})^{2} $$

where *σ, k*_b_, *T*, *h*, *R*, and s refer to the reaction path degeneracy, Boltzmann constant, temperature in Kelvin, Planck constant, ideal gas constant, and the distance along the minimum energy reaction path (MEP) in isoinertial coordinates, respectively.

Global chemical reactivity descriptors^45^ have been calculated using the energies of the highest occupied and lowest unoccupied molecular orbitals (HOMO and LUMO), respectively, to better comprehend the reactivity and stability of the structures under study. Accordingly, for B3LYP/6-31G(d,p) in the gas phase, the ionization potential (IP), electron affinity (EA), absolute hardness (η), softness (S), and electronegativity (χ) were calculated. The following formulas have been used to calculate the global chemical reactivity descriptors:

4 $$ {\text{IP}} = - {\text{E}}_{{{\text{HOMO}}}} $$

5 $$ {\text{EA}} = - {\text{E}}_{{{\text{LUMO}}}} $$

6 $$ {\upeta } = \left( {{\text{E}}_{{{\text{LUMO}}}} - {\text{E}}_{{{\text{HOMO}}}} } \right)/2 $$

7 $$ {\text{S}} = 1/2\eta $$

8 $$ \chi = - \left( {{\text{E}}_{{{\text{LIUMO}}}} + {\text{E}}_{{{\text{HOMO}}}} } \right)/2 $$

Nucleus-independent chemical shift (NICS) computations have been performed for each compound in the gas phase at the same level of optimization (B3LYP/6-31G(d,p)). Particularly, the computed NICS values are NICS(0)iso, which refers to the absolute isotropic shielding at the center of the ring; NICS(0)zz, which is the total contribution to the orthogonal to the plane component of the NICS tensor, assuming the molecule lies in the xy-plane; as well as NICS(1)iso and NICS(1)zz, which are analogue indices computed at 1 A above the center of the ring^46,47^. The geometry-based index harmonic oscillator measure of aromaticity (HOMA)^48,49^, which makes use of bond lengths in accordance with Krygowski's^48^ instructions, is another criterion of aromaticity. Electrostatic potential (ESP) surfaces have been derived from the B3LYP/6–31G(d,p) calculation.

UV–vis spectra for the investigated structures were computed at TD-DFT PBE^50,51^ (TD-PBE/6–311+G(d,p)) using the SMD method at the B3LYP/6-31G(d,p) optimized gas-phase geometry in acetonitrile. The electron excitations of 2OHPhAZ were precisely determined by the Perdew-Burke-Ernzerhof technique (PBE)^22^. GaussSum program^52^ was used to simulate the ultraviolet–visible (UV–vis) spectra. Instead of focusing on the canonical orbitals, natural transition orbitals (NTOs)^53^ were estimated for each electron excitation. We create the frontier orbitals and NTOs using Chemcraft. Additionally, we use the optimized gas-phase geometry to calculate the NMR spectra of the various tautomers and rotamers relative to the 13C and 1H isotropic chemical shielding of tetramethylsilane (TMS) at the B3LYP/6-31G(d,p) in chloroform using the gauge-independent atomic orbital (GIAO) method^54,55^.

We concentrated on the hyper-Rayleigh scattering (*β*_*HRS*_) and depolarization ratio (DR) among second-order NLO characteristics^56^, and the complete equations for calculating the magnitude of the total dipole moment μ_*tot*_, the average polarizability α_*tot*_ and the first hyperpolarizability *β*_*tot*_, using the *x*, *y* and *z* components are as follows:^57,58^

9 $$ \mu = \, \left( {\mu^{{2}}_{x} + \mu^{{2}}_{y} + \mu^{{2}}_{z} } \right)^{{{1}/{2}}} $$

10 $$ \left\langle \alpha \right\rangle = {1}/{3}(\alpha_{xx} + \alpha_{yy} + \alpha_{zz} ) $$

11 $$ \Delta \alpha = \, \left( {\left( {\alpha_{xx} - \alpha_{yy} } \right)^{{2}} + \, \left( {\alpha_{yy} - \alpha_{zz} } \right)^{{2}} + \, \left( {\alpha_{zz} - \alpha_{xx} } \right)^{{2}} /{2}} \right)^{{{1}/{2}}} \left\langle \beta \right\rangle_{tot} \; = \;\left[ {\left( {\beta_{xxx} + \beta_{xyy} + \beta_{xzz} } \right)^{{2}} + \, \left( {\beta_{yyy} + \beta_{xxy} + \beta_{yzz} } \right)^{{2}} + \, \left( {\beta_{zzz} + \beta_{xxz} + \beta_{yyz} } \right)^{{2}} } \right]^{{{1}/{2}}} $$

12 $$ \beta \left( {_{HRs} } \right) \, = \sqrt {\beta^{2} zzz + \beta^{2} } zxx $$

13 $$ {\text{DR}} = \frac{{\beta^{2} zzz}}{{\beta^{2} zxx }} $$

## Results and discussion

### Geometries optimization

According to previous research, the M06-2X and B97XD functionals are extremely accurate in predicting the stability of tautomers and rotamers trend^59–61^. The same order of stability was also created by B3LYP/6–31+G(d,p) as obtained by the ab initio multi-level CBS-QB3 approach^62^. Consequently, the structures will be discussed at B3LYP/6-31G(d,p), and energies will be discussed at M06-2X/6–311++G(2d,2p), B97XD/6–311++G(2d,2p), and CCSD(T)/6–311++G(2d,2p).

**Figure 2:**
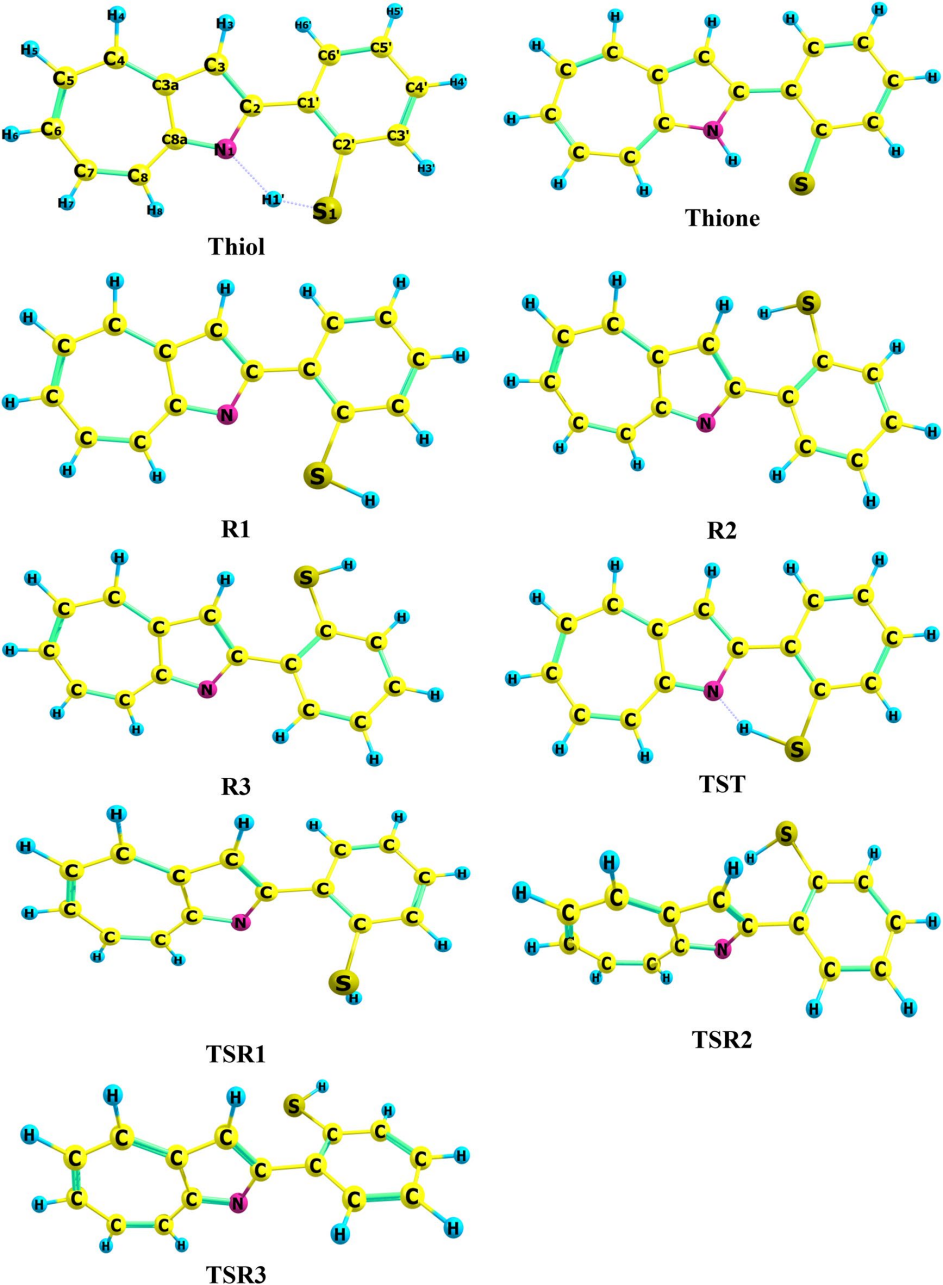
Optimized structures of the 2-(2-Mercaptophenyl)-1-azaazulene tautomer and rotamers with their corresponding transition states at B3LYP/6-31G d,p) in the gas phase.

2-(2-Mercaptophenyl)-1-azaazulene could exist as thione-thiol tautomerism based on the movement of hydrogen atom among nitrogen and sulfur, and three rotamers form R1, R2, and R3. The optimized geometries of the studied tautomeric and rotameric forms and their corresponding inter-conversion transition states are shown in Fig. 2, and their geometrical parameters are listed in Table 1 at the B3LYP/6-31G(d,p) level. Also, Table S1 in the supplementary information (SI) reported their coordinates. The rotamer R1 is formed by rotating the OH group of thiol, while R2 and R3 are formed by rotating the phenyl ring of thiol and R1, respectively. To the best of our knowledge, there are no theoretical and experimental reports on the geometry of the titled compound conformers in the literature for comparison. The calculated geometrical parameters are in good agreement with the corresponding values reported for a similar compound^22^.

**Table 1:** Calculated bond lengths (Å) and angles (in °) of 2-(2-Mercaptophenyl)-1-azaazulene tautomers and rotamers at the B3LYP/6-31G(d,p) in the gas phase.

Bond/angles ^a^	Thiol	Thione	R1	R2	R3
S1-H1'	1.371	2.015	1.355	1.347	1.347
N1-H1'	1.808	1.056	4.114	3.851	5.690
C2'-S1	1.779	1.735	1.790	1.794	1.794
N1-C2	1.364	1.375	1.357	1.365	1.366
C1'–C2'	1.424	1.449	1.420	1.415	1.416
C2–C1'	1.470	1.433	1.465	1.475	1.473
C2'–C3'	1.407	1.428	1.406	1.402	1.403
C8a–N1	1.349	1.359	1.348	1.346	1.344
N1H1'S1	143.3	142.2	6.7	115.2	25.2
H1'S1C2'	94.4	87.5	93.3	97.6	95.4
N1C2C1'	122.9	122.7	120.7	118.7	118.6
S1C2'C1'	126.1	126.5	122.4	125.5	121.3
S1C2'C3'	114.9	117.3	118.9	114.7	119.1
N1C2C1'C2'	–13.9	0.0	13.1	151.5	156.2
C2C1'C2'S1	0.6	0.0	0.4	–3.5	– 3.8
C1'C2'S1H1'	7.4	0.0	171.6	–25.6	– 175.1
H1'N1C2C1'	13.5	0.0	– 13.7	– 103.5	– 58.8

^a^Atom numbering is given in Fig. 2.

The thione form in the gas phase shows that it is almost planar with a dihedral angle (N1C2C1'C2') of 0.0^o^, but in the case of the thiol form, the terminal phenyl ring is out of plane with a dihedral angle (N1C2C1'C2') of—13.9°. The thione-thiol tautomerization occurs via an IPT reaction. The intramolecular hydrogen bond generates a six-membered ring in both the thiol and thione tautomers. The intramolecular H-bonding is between the S1-H1’ donor and N1 acceptor (1.808 Å) of the thiol tautomer, but in the case of the thione tautomer, the interaction is between the N–H donor and the thiophenol S1 acceptor, where the hydrogen-bond lengths are about 2.015 Å. In the IPT, the C2′-S1 bond length decreases from 1.779 in the thiol form to 1.735 Å in the thione form. Also, the calculated S-H1'…N hydrogen-bond angles decrease from 143.3° in the thiol form to 142.2° in the thione form.

The thiol-thione tautomer passes through the transition state (TST) (see Fig. 2). The extracted observations of the proton shift process include: (1) The S1′-H1′ bond length of 0.968 Å in thiol elongated by 0.203 Å in the TST and eventually separated to release proton; (2) The proton then migrated and approached N1 with a distance of 1.329 Å in the TST before the formation of the N1-H1’ bond (1.056 Å) in thione; (3) In concerted processes, the thiol S1C2’C1’ and N1 C2 C1’ angles closed up by 6.2° and 1.7°, respectively, in the TST and finally settling at 126.5◦ and 122.7◦, respectively, in thione.

In the process of rotamerization, the observed slightly increased C2'-S1 bond distance for R2 and R3 (1.794 Ǻ) compared to R1 (1.789 Ǻ) is due to the decrease in double bond character in R2 and R3 because there is no conjugation of the sigma lone pair in R2 and R3. Additionally, there is no hydrogen bond in the R1 rotamer, and the donor–acceptor distance of 2.773 Ǻ is smaller than the corresponding distance in thiol of 3.021 Ǻ. The S1 − H1' bond length in TSR1 and R1 is similar but shorter than that in thiol by 0.02 Å due to the presence of HB in the latter structure. The H1' − S1 − C2' angle is rotated by 3.4° from R1. The donor–acceptor distance in TSR1 is elongated to 3.249 Å. The calculated dihedral angles show that the planarity of rotamers is distorted (Table 1). The non-rigid structure of 2-(2-Mercaptophenyl)-1-azaazulene tautomers and rotamers gives them the ability to fit with receptors in biological systems.

### Tautomerism and thermodynamic properties

This section deals with the study of equilibrium between the studied tautomers and rotamers using the DFT/M06-2X and ωB97XD in comparison to the more accurate CCSD (T) calculations. Table 2 presents the theoretically computed corrected relative energies (Δ*E*), Gibbs free energies (ΔG), and enthalpies (ΔH) of the five tautomers and rotamers at the ωB97XD/6–311++G(2d,2p), M06-2X/6–311++G(2d,2p), and CCSD(T)/6–311++G(2d,2p) levels in the gas and ethanol phases. For tautomerization, it is found that the thiol form is more stable than the thione form in the gas phase. The relative energy difference between the thiol and thione in the gas phase is 3.30 and 5.30 kcal/mol at ωB97XD and CCSD(T), respectively. The thiol stability in the gas phase is expected by having a lower and stronger IHB than thione and by having the higher aromaticity of the phenyl ring that has been broken by the formation of the thione form (as will be discussed in Section "Aromaticity").

**Table 2:** Relative zero-point corrected energies (Δ*E*^0^), relative Gibbs free energies (ΔG_298_), enthalpies (ΔH_298_) in kcal/mol, and dipole moments (µ; in Debye) for the 2-(2-Mercaptophenyl)-1-azaazulene tautomers, rotamers, and their transition states in the gas phase and ethanol.

Parameters	Thiol	Thione	R1	R2	R3	TS_T_	TS_R1_	TS_R2_	TS_R3_
M06-2X^a^ (Gas phase)									
Δ*E*^0^	0.00	2.89	0.94	0.68	0.77	2.10	4.38	2.53	3.57
ΔG_298_	0.00	3.34	0.73	0.69	0.69	2.65	4.37	3.11	4.16
ΔH_298_	0.00	2.76	1.02	0.87	0.97	1.82	4.33	2.37	3.42
µ	4.67	8.71	2.53	3.13	1.91	6.93	2.62	3.67	1.91
ωB97XD^a^ (Gas phase)									
Δ*E*^0^	0.00	3.30	1.90	1.46	1.88	2.47	4.75	2.41	3.58
ΔG_298_	0.00	3.75	1.69	1.47	1.80	3.02	4.75	2.99	4.16
ΔH_298_	0.00	3.16	1.98	1.65	2.08	2.19	4.70	2.25	3.42
µ	4.83	9.03	2.66	3.28	1.99	7.10	2.72	3.79	2.65
CCSD(T)^a^ (Gas phase)									
Δ*E*^0^	0.00	5.30	1.43	0.76	1.05	3.63	3.71	1.55	2.68
ΔG_298_	0.0	5.71	0.94	0.77	0.98	4.18	3.71	2.13	3.26
ΔH_298_	0.00	5.12	1.22	0.95	1.26	3.35	3.67	1.39	2.52
µ	5.01	10.14	2.09	3.48	2.09	7.56	2.93	3.99	2.79
M06-2X^a^ (Ethanol)									
Δ*E*^0^	4.34	0.00	4.27	4.90	4.25	4.12	7.58	5.63	5.80
ΔG_298_	5.41	0.00	5.57	6.01	5.30	5.65	8.67	7.08	7.55
ΔH_298_	4.36	0.00	3.80	5.15	4.52	3.83	7.58	5.58	5.66
µ	7.77	16.00	4.31	5.54	3.61	9.70	4.63	6.36	4.26
ωB97XD^a^ (Ethanol)									
Δ*E*^0^	4.50	0.00	5.42	5.49	5.30	4.39	7.93	5.40	5.87
ΔG_298_	5,57	0.00	6.71	6.61	6.34	5.92	9.02	6.86	7.62
ΔH_298_	4.53	0.00	4.94	5.74	5.56	5.00	7.93	5.36	5.74
µ	7.97	16.31	4.49	5.76	3.76	9.89	4.79	6.55	4.40

^a^Method/6–311++G(2d,2p)//B3LYP/6-31G(d,p).

In the rotamerization process, the order of stability of the rotameric structures is R2 > R3 > R1. Rotamer R2 is slightly more stable than R3 and R1 by about 0.42 and 0.44 kcal/mol at ωB97XD/6–311 + +G(2d,2p), respectively. R1 is the least stable rotamer at all calculated levels. The stability of R2 and R3 can be attributed to the possible intramolecular H bonding interaction of the C6′–H6′…N1 as shown in Fig. 1. The slightly higher stability of R3 than R2 can be due to the anti-position present between the S1-H1’ group and the nitrogen of the azaazulene ring, given the possibility of H3 interaction with the sulfur atom.

Mezey et al.^63,64^ proposed that any species with a higher energy than 10 kcal mol^−1^ above the most stable form would not exist in any appreciable concentration. So, we can conclude that all the studied tautomers and rotamers of 2-(2-Mercaptophenyl)-1-azaazulene can be observed and exist experimentally by having a low difference in the relative energies (Table 2). A chemical equilibrium between tautomers and rotamers is characterized by the equilibrium constant (*K*) that can be calculated using:

14 $$ K = {\text{ e}} -^{{(\Delta {\text{G}}/{\text{RT}})}} $$

where the gas constant (R) is 1.987*10^–3^ kcal/mol and the temperature (*T*) is 298.15 K and the ΔG is the difference between the Gibbs free energies of a given tautomer or rotamer relative to thiol. Table 3 collects the *K* values for the four possible equilibrium reactions in the gas phase and ethanol at ωB97XD/6–311 ++G(2d,2p) and CCSD(T)/6–311++G(2d,2p).

**Table 3:** Equilibrium constants for 2-(2-Mercaptophenyl)-1-azaazulene tautomers and rotamers in gas phase and ethanol at ωB97XD^a^/6–311++G(d,p) basis set and CCSD(T)/6–311++G(d,p).

Equilibrium reaction		ωB97XD^a^		CCSD(T)^a^
		Gas phase	Ethanol	Gas phase
Thiol $\mathop \leftrightarrow \limits^{{{\text{k}}1}}$ thione	*K*_*1*_	0.002	1.208 × 10^4^	6.551 × 10^–5^
Thiol $\mathop \leftrightarrow \limits^{{{\text{k}}2}}$ R1	*K*_*2*_	0. 058	0.146	0.206
Thiol $\mathop \leftrightarrow \limits^{{{\text{k}}3}}$ R2	*K*_*3*_	0.084	0.172	0.272
Thiol $\mathop \leftrightarrow \limits^{{{\text{k}}4}}$ R3	*K*_*4*_	0.048	0.270	0.193

^a^Method/6–311++G2d,2p)//B3LYP/6-31G(d,p).

In accordance with the highest stability of thiol among the other tautomers and rotamers in the gas phase, *K*_1_ has the lowest values. This implies the presence of thiol alone in the gas phase. The values of *K*_2_, *K*_3_ and *K*_4_ (see Table 3) indicate that the equilibrium is effective and the rotamers with the thiol are present in comparable quantities in both the gas phase and ethanol. The calculated rate constant for the studied transformation with VTST and wigner tunneling is given in Table 4. The results show that the tautomerization transformation has a higher rate and a higher tunneling correction effect than rotamerization in the gas phase during the applied temperature range. The rate of rotamerization seems to slightly increase as the temperature is raised.

**Table 4:** Lists the VTST rate constant and wigner tunneling of the tautomerization and rotamerization conversion processes over a temperature range 270–320 K at B3LYP/6-31G(d,p) in the gas phase.

*T* (K)	Thiol → thione (TS_T_)		Thiol → R1 (TS_R1_**)**		Thiol → R2 (TS_R2_**)**		Thiol → R3 (TS_R3_)	
	$\chi (T)$	$k_{{}}^{CVT}$	$\chi (T)$	$k_{{}}^{CVT}$	$\chi (T)$	$k_{{}}^{CVT}$	$\chi (T)$	$k_{{}}^{CVT}$
270	2.23	1.64E + 12	1.06	4.72E + 06	1	1.66E + 07	1	3.78E + 06
280	2.14	1.67E + 12	1.05	8.05E + 06	1	2.60E + 07	1	6.23E + 06
290	2.06	1.71E + 12	1.05	1.32E + 07	1	3.95E + 07	1	9.94E + 06
300	1.99	1.74E + 12	1.05	2.11E + 07	1	5.84E + 07	1	1.54E + 07
310	1.93	1.77E + 12	1.04	3.26E + 07	1	8.42E + 07	1	2.31E + 07
320	1.87	1.80E + 12	1.04	4.90E + 07	1	1.19E + 08	1	3.40E + 07

The heterocyclic tautomerism is very sensitive to the solvent nature. Therefore, more efforts have been made to study the effect of solvents such as ethanol on the stability of the tautomers and rotamers using the same levels of theory. Energy results obtained from these calculations are collected in Table 2. In contrast to the gas phase, the higher stability of the thione form than thiol in ethanol has been observed at all levels of calculations. The stability order is reversed in ethanol, and the thione becomes more stable relative to thiol by 4.50 kcal/mol at the ωB97XD/6–311++G(2d,2p) level (Table 2). Also, the higher value of the equilibrium constant (1.208*10^4^) emphasized the higher stability of thione in the ethanol solvent (Table 3).

From both theoretical and experimental points of view, the thione-thiol tautomer reaction presents challenges. Computational studies of sulfur-containing molecules often show large basis set effects^65,66^. There is evidence that the thione-thiol tautomeric equilibrium between 2-pyridinethiol (2SH) and 2-pyridinethione (2S) also strongly depends on the environment. 2S is generally believed to be more stable in polar solvents^67,68^ while 2SH has high stability in the gas phase and non-polar solvents^69,70^. The stability order of thione-thiol tautomers of 2-(2-Mercaptophenyl)-1-azaazulene agrees with the predominance of 2-pyridinethione (thione form) over 2-pyridinethiol (thiol form) in polar solvents^67,68^.

The calculated dipole moment values for all tautomers and rotamers at the ωB97XD/6–311++G(2d,2p) level of theory in the gas and ethanol phases are given in Table 2. It can be noted that thione has the highest polarity. As a result, it has a significant impact on the stability order in ethanol as a polar solvent. Obviously, a larger dipole moment leads to greater stabilization. As can be seen in Table 2, the thione has 16.31 D, while the thiol, R1, R2 and R3 have 7.97, 4.49, 5.76 and 3.76 D, respectively, at ωB97XD/6–311++G(2d,2p) level of theory in the ethanol phase.

Comparing the previous findings with those of the ethanol phase, it is observed that the relative energy difference is positive for all the tautomers and rotamers. This result suggests that the presence of ethanol helps stabilize them, with a clear stabilization of five tautomers and rotamers. For the given tautomers and rotamers, the results show that the thiol form has the lowest formation enthalpy values (Table 2) in the gas phase. This means that it is more stable than the thione form. The lower positive reaction enthalpies mean that the equilibrium reaction is an endothermic one.

### Chemical reactivity

#### Theory of frontier molecular orbitals

HOMO and LUMO orbitals play a fundamental role in the qualitative interpretation of chemical reactivity^71^. The energy gap between HOMO and LUMO frontier orbitals is one of the significant characteristics of molecules and plays an important role in electric properties, electronic spectra, and photochemical reactions. The HOMO–LUMO energy gap helps characterize the chemical reactivity and kinetic stability of the molecule^72^. A molecule with a high energy gap (Δ*E*) is less polarizable and is generally associated with low chemical reactivity and high kinetic stability^73^. Table 5 contains the energies of the HOMO LUMO boundary orbitals and the HOMO–LUMO energy gaps in gas and ethanol phases. The energy gap values decrease in the ethanol (Table 5) compared with those obtained in the gas phase. Therefore, the tautomers and rotamers are more reactive in ethanol. The obtained value of the thione tautomer (Δ*E*_gap_ = 1.87 and 0.08 eV) is smaller than those obtained with thiol (Δ*E*_gap_ = 2.98 and 0.12 eV) in gas and ethanol, respectively. So, thione is the most polarizable and has the highest interactions for intramolecular charge transfer. The large energy gap of the thiol confirms the high stability of the thiol tautomer. The rotamers (R1-R3) are hard molecules and have the least ability to polarize because they have a higher energy gap than tautomers. R3 has the largest value of the energy gap, 3.44 and 0.13 eV in the gas phase and ethanol, respectively.

**Table 5:** Global chemical descriptor (eV) of the studied structures at B3LYP/6-31G (d,p) in the gas phase and ethanol.

	*E*_*HOMO*_	*E*_*LUMO*_	*E*_*g*_	*IP*	*EA*	*Χ*	*η*	*S*
Tautomers/rotamers (Gas phase)								
Thiol	–5.42	–2.44	2.98	5.42	2.44	3.93	1.49	0.75
Thione	–4.70	–2.83	1.87	4.70	2.83	3.77	0.93	0.47
R1	–5.49	–2.25	3.24	5.49	2.25	3.87	1.62	0.81
R2	–5.70	–2.33	3.37	5.70	2.33	4.01	1.68	0.84
R3	–5.67	–2.23	3.44	5.67	2.23	3.95	1.72	0.86
(Ethanol phase)								
Thiol	–0.204	–0.084	0.129	0.204	0.085	0.144	0.06	0.03
Thione	–0.178	–0.094	0.084	0.178	0.095	0.137	0.04	0.02
R1	–0.207	–0.081	0.125	0.207	0.081	0.144	0.06	0.03
R2	–0.211	–0.082	0.129	0.211	0.082	0.146	0.06	0.03
R3	–0.212	–0.081	0.130	0.211	0.081	0.146	0.07	0.03

#### Global indices of reactivity

The study of the global reactivity of molecules is based on the calculation of global indices deduced from electronic properties. The global indices of the reactivity in the gas phase and ethanol of the studied conformers are recorded in Table 5. Chemical hardness (*η*) and global softness (*S*) express the resistance of a system to a change in its number of electrons. In a given series of molecules, when *η* is weak, the molecule is called soft and when it is high, the molecule is called hard. This is quite the opposite of softness, which evolves in the opposite direction of hardness^74^. The value of the chemical hardness of thione is the lowest, where* η* = 0.93 and 0.04 eV in the gas phase and ethanol, respectively. Also, we note that the thione form has a lower electronegativity value (*χ* = 3.77 eV) than other tautomers and rotamers in the gas phase, which supports the stability of thiol in the gas phase.

#### Electrostatic potential (ESP)

The electrostatic potential (ESP) surface is another useful tool to understand the chemical reactivity of a molecule. The ESP study shows that H-donor and H-acceptor properties of molecules are revealed by positive and negative regions, respectively^75^.

**Figure 3:**
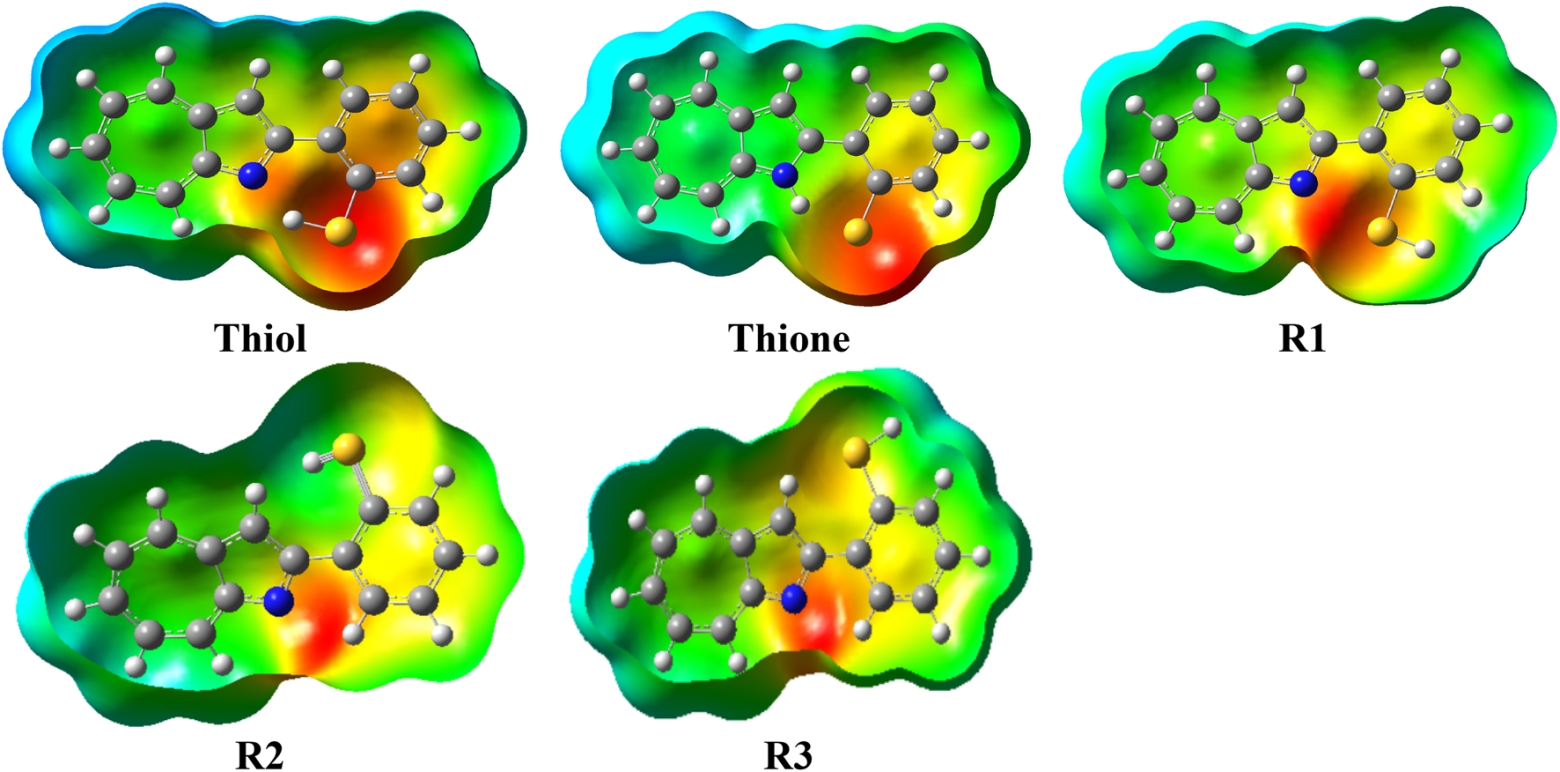
Molecular ESP surfaces of the investigated structures at the B3LYP/6-31G(d,p) in gas phase.

By using ESP maps, the charged areas of the molecule can be shown. In ESP maps, the regions with the highest negative electrostatic potential are shown in red, while those with the most positive electrostatic potential are shown in blue. Figure 3 shows the ESP surfaces of the examined structures derived using the B3LYP/6-31G (d,p). The ESP of thiol, thione, and R1 shows that a substantial amount of negative charge is localized on the S atom, with just a tiny amount of negative charge localized on the sulfur atom for R1.

As a result, the S atom is believed to have the highest electron donation ability towards the formation of a H-bond or metal ions. The blue colour around the H atoms indicates their positive charge. For R2 and R3 the negativity charge becomes localized on the N atom. Therefore, R2 and R3 have a high tendency to bind with metal ions on the N side.

#### Aromaticity

The aromaticity of organic compounds is one of the most important characteristics related to their specific chemical reactive structure^76^. In this study, we used some aromaticity indicators that depend on geometry. HOMA is one of the best ways to describe the change in aromaticity^48,49^. When HOMA is unity^66^, then the compound is completely aromatic, while if HOMA equals zero (0), the compound or ring is completely non-aromatic. More aromatic rings have more π-electrons delocalized, and this is indicated by rings with higher HOMA values. HOMA can be calculated according to the following equation:

15 $$ HOMA = 1 - \frac{1}{n}\mathop \sum \limits_{j = 1}^{n} \alpha_{i} \left( {R_{opt,i} - R_{j} } \right)^{2} $$

where α_i_ is a normalization constant (α_CC_ = 257.7 and α_CN_ = 93.52) and n is the total number of bonds in the molecule. The optimized bond length, or R_opt_, is 1.388 Å for a C–C bond and 1.334 Å for a C-N bond. The results of HOMA calculations at the B3LYP/6-31G(d,p) level in the gas and ethanol phases are shown in Table 6. Evidently, Table 6's HOMA results offer a good match with the relative energies and the structure of the thiol-thione tautomer in both gas and ethanol solvent. The strength of HB of thiol in the gas phase can be attributed to the highest value of HOMA (0.89) of the thiophenyl ring. Through the formation of thione tautomer, the five- and six-membered rings exhibit reasonably higher and lower delocalization, with HOMA values of 0.61 and 0.67, respectively. The five-membered ring's lower HOMA values in the thiol form (0.48) compared to the thione (0.61) tautomer indicate that its electron delocalization to the IHB ring in the thiol form is involved. With a slightly higher HOMA value than the five-membered ring due to tautomerization, the seven-membered ring indicates a greater aromatic character. From the HOMA values of all rings, thiol seems to have strong π-stacking ability, hence having more aromaticity and stability in gas phase. In ethanol, the aromaticity of the five- and seven-membered rings of thione is increased (0.66 and 0.76, respectively), which raises the summation of HOMA. Thus, the aromaticity and stability of thione are noticed in ethanol solvent. The comparison of HOMA values for the thiophenyl ring and five-membered ring for R1 in the gas phase (0.92 and 0.44), R2 (0.94 and 0.46) and R3 (0.93 and 0.47) seems to suggest a stronger delocalization and the higher stability of the R2 structure. A slight change observed in ethanol preserves the higher stability of R2 and R3 than R1.

**Table 6:** The NICS(1)_zz_ (in ppm) and HOMA index of the studied tautomers and rotamers calculated in gas phase and ethanol at the B3LYP/6-31G(d,p). Five- and seven-membered rings of azaazulene and phenyl rings are presented in Fig. 2.

Compound		Gas-phase		Ethanol	
		NICS(1)_zz_	HOMA	NICS(1)_zz_	HOMA
Thiol	Five-membered ring	– 38.74	0.48	– 38.52	0.54
	Seven-membered ring	– 20.23	0.65	– 20.62	0.69
	Six-membered ring	– 18.96	0.89	– 19.52	0.90
∑ HOMA			2.02		2.13
Thione	Five-membered ring	– 26.27	0.61	– 29.40	0.66
	Seven-membered ring	– 11.63	0.69	– 18.06	0.76
	Six-membered ring	– 14.57	0.67	– 17.29	0.77
∑ HOMA			1.97		2.19
R1	Five-membered ring	– 38.63	0.44	– 38.24	0.49
	Seven-membered ring	– 20.32	0.62	– 20.54	0.66
	Six-membered ring	– 21.83	0.92	– 22.21	0.92
∑ HOMA			1.98		2.07
R2	Five-membered ring	– 34.64	0.46	– 34.09	0.53
	Seven-membered ring	– 19.89	0.64	– 20.17	0.68
	Six-membered ring	– 19.75	0.94	– 19.86	0.93
∑ HOMA			2.04		2.14
R3	Five-membered ring	– 37.60	0.47	– 37.00	0.53
	Seven-membered ring	– 20.85	0.64	– 21.11	0.67
	Six-membered ring	– 22.46	0.93	– 22.58	0.93
∑ HOMA			2.04		2.13

Another aromaticity investigation that can be assessed through magnetic criteria is the NICS^46,47^. That can be defined as the negative value of the absolute magnetic shielding calculated at some selective points. The more negative the NICS values, the more aromatic the ring. The NICS(0) and NICS(1) values are computed at the center and 1A˚ above the aromatic ring, respectively. Aromatic systems possess negative NICS values, as that indicates the presence of an induced diatropic ring current. Positive NICS values refer to paratropicity and are present in antiaromatic systems. Very small NICS values refer to nonaromatic systems. The NICS indices calculated as a single point at the B3LYP/6–31 G(d,p) level in the gas phase are given in Table 6. According to previous studies of NICS calculation^46,47,77–79^, the five-membered ring is more aromatic than the six-membered ring followed by the seven-membered ring. As can be seen from Table 6, the pyrrole ring is more aromatic than benzene, and the aromaticity is less in the seven-membered ring. In the gas phase, the thiophenyl ring of thiol exhibits a larger negative NICS(1)zz value (-18.96 ppm) than thione (NICS(1)zz (-14.57)). In the presence of a polar solvent like ethanol, very minute variations in magnetic indices of aromaticity are seen. Due to the absence of IHB production and an increase in π-electron delocalization, the investigated rotamers exhibit somewhat larger NICS(1)zz values of the thiophenyl ring than thiol.

### NMR analysis

The high correlation between the experimental^21^ and theoretical^22^ NMR chemical shifts of 2-(2-hydroxyphenyl)-1-azaazulene (2OHPhAZ) at the B3LYP/6-31G(d,p) level that has been previously recorded^22^ imparts trust in the computational techniques utilized. The calculated ^1^H and ^13^C NMR chemical shifts $\delta$(ppm) values for the 2-(2-Mercaptophenyl)-1-azaazulene tautomers and rotamers calculated in CHCl_3_ at the B3LYP/6-31G (d,p) level of theory are listed in Table 7. For the ^1^H NMR chemical shift, the hydrogen atoms bonded to carbon atoms in aromatic rings, ranging from 6 to 8 ppm^80–82^. The thione NH1 proton and the thiol SH1 proton have high chemical shifts (high downfield shift/low-field proton signal) at $\delta$ 18.4 and 13.6 ppm, respectively. While for rotamers, the SH_1_ proton was assigned at lower chemical shifts in the range of $\delta$ 3.9 to 4.6 ppm. This is due to the presence of HB in the thiol and thione structures and the absence of HB in the structures of rotamers. The azaazulenyl ring and phenyl ring protons in the studied compounds showed a low chemical shift in the range of 7.2 to 8.9 ppm.

**Table 7:** ^13^C and ^1^H NMR chemical shifts (in ppm) calculated for C2' and H1-S/N at the B3LYP/6-31G(d,p) level of theory of 2-(2-Mercaptophenyl)-1-azaazulene tautomer and rotamers using the GIAO method in CHCl_3_.

	C2'	H1- S1/N
Thiol	143.35	13.58
Thione	135.12	18.44
R1	127.46	4.02
R2	123.77	4.55
R3	124.22	3.93

The computed ^13^C NMR chemical shifts for 2-(2-Mercaptophenyl)-1-azaazulene tautomers and rotamers are in the region 104.0–173.8 ppm (in CHCl_3_). The highest chemical shift values given in thiol are C2 (163.4 ppm) and C2' in thione (173.8 ppm). This low field shift is due to the fact that C2 and C2' are bonded to the high electronegativity N and S atoms and the high polarity of the NH and C = S groups in thiol and thione, respectively. Similarly, C8a has a high chemical shift (greater than 140 ppm) for thiol and all the studied rotamers by connecting to the N atom. The highest chemical shifts of C2' and H1 in the studied thione correlated well with the highest chemical shift of C2' and H1 in the keto of 2OHPhAZ ($\delta$ 171.3 and 19.4 ppm, respectivel)^22^. The calculated chemical shift of the thiocarbonyl group (C = S) (173.8 ppm) is in good agreement with the reported chemical shifts of 169.9 and 177.7 ppm for 3-Hydroxy-2-methyl-4-p-pyridinethione^83^ and 5-(3-pyridyl)-4H-1,2,4-triazole-3-thione^84^, respectively.

### UV–Vis spectral analysis

**Figure 4:**
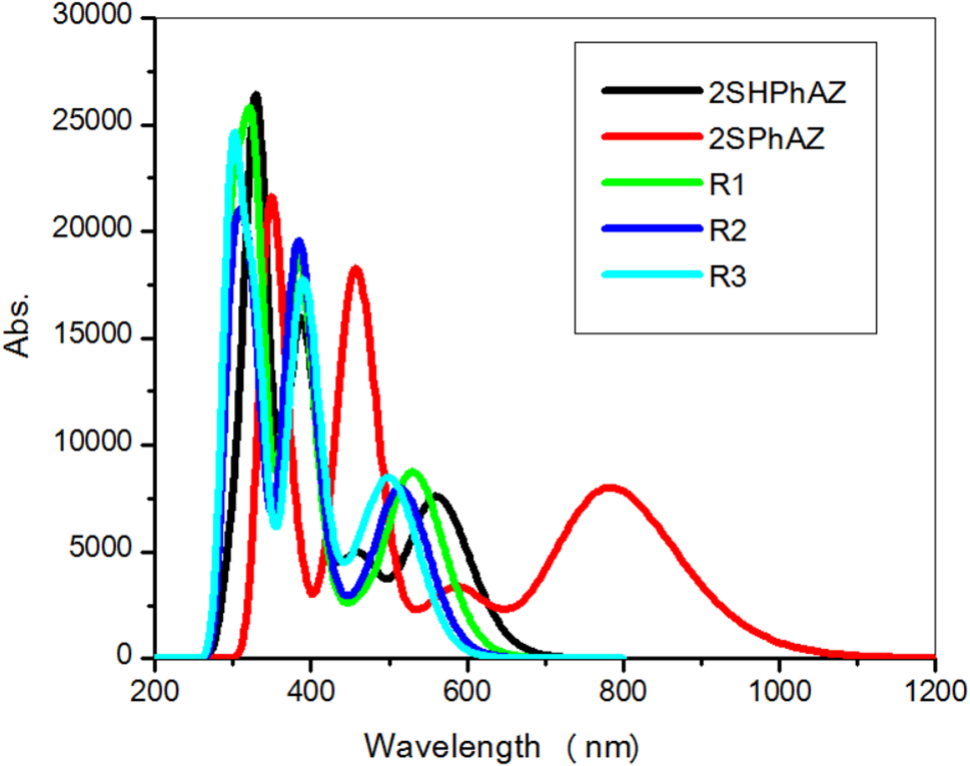
The simulated UV/Vis absorption spectra for the thiol, thione and rotamers of 2-(2-Mercaptophenyl)-1-azaazulene at TD-PBE/6-311+G (d,p).

We have found that our previous calculation of 2OHPhAZ^22^ using TDDFT-PBE/SMD with a 6–311+G(d,p) basis set level exhibits better quantitative agreement regarding the first and second maximum excitation peaks with the available experimental data^21^ than other levels^22^. The calculated UV–vis parameters of the intense peaks in acetonitrile for the studied tautomers and rotamers with the TD-PBE method are presented in Table 8. The calculated UV–vis spectra for all the studied compounds are given in Fig. 4. The thione form is accompanied by a large red shift (extended to 1000 nm), followed by the thiol form (extended to 700 nm), and the lower shift has been found with the rotamers (extended to 600 nm), as depicted in Fig. 4. As can be seen in Table 5, the energy differences between HOMOs and LUMOs are 2.98 eV (for thiol), 1.87 eV (for thione), 3.24 eV (for R1), 2.33 eV (for R2) and 2.23 eV (for R3) in the gas phase. As expected from the lower *E*_g_ value of thione, the thione maxima in the electronic absorption spectra are shifted bathochromically by 16, 20, 22 and 47 nm in comparison with thiol, R1_,_ R2, and R3, respectively.

**Table 8:** Excitation energies (eV) at (TD-PBE-SMD, acetonitrile)/6–311+G(d,p)//B3LYP/6-31G(d,p), oscillator strengths (*f* > 0.1), and their transition characters for 2-(2-Mercaptophenyl)-1-azaazulene tautomers and rotamers.

Compound	State	*E*^a^	*F*	Assignment^b^
Thiol	1	2.21(561)	0.1041	H → L (91%)
	5	3.22(385)	0.1554	H-3 → L (35%), H-2 → L (42%), H-1 → L + 1 (17%)
	6	3.73(332)	0.3052	H-2 → L (11%), H-2 → L + 1 (17%), H-1 → L + 1 (50%)
Thione	1	1.58 (783)	0.1103	H → L (94%)
	5	2.72(456)	0.2522	H-2 → L (91%)
	9	3.57 (348)	0.2752	H-3 → L (38%), H-2 → L + 1 (38%)
R1	1	2.33 (531)	0.1186	H → L (89%)
	5	3.25 (382)	0.2400	H-2 → L (54%), H-1 → L + 1 (27%), H → L + 1 (10%)
	6	3.78 (328)	0.2639	H-2 → L + 1 (24%), H-1 → L + 1 (39%), H → L + 1 (12%)
	10	4.21 (294)	0.1658	H-4 → L (10%), H-1 → L + 3 (17%), H → L + 3 (55%)
R2	1	2.41 (515)	0.1077	H → L (89%)
	4	3.25 (382)	0.2012	H-2 → L (41%), H-1 → L + 1 (19%), H → L + 1 (23%)
	6	3.81 (326)	0.1836	H-2 → L + 1 (37%), H-1 → L + 1 (32%), H → L + 1 (10%)
	8	4.05 (306)	0.1418	H-4 → L (11%), H-2 → L + 1 (27%), H-1 → L + 1 (10%), H → L + 2 (34%)
	10	4.22 (294)	0.1025	H-5 → L (14%), H-3 → L + 1 (27%), H → L + 3 (33%)
R3	1	2.45 (506)	0.1034	H-1 → L (12%), H → L (81%)
	3	3.13 (396)	0.1283	H-2 → L (44%), H → L + 1 (45%)
	4	3.22 (385)	0.1138	H-2 → L(31%), H-1 → L + 1 (22%), H → L + 1 (35%)
	6	3.78 (328)	0.2062	H-2 → L + 1 (32%), H-1 → L + 1 (35%), H → L + 1 (12%)
	8	4.11 (301)	0.2557	H-2 → L + 1 (13%), H → L + 2 (64%)

^a^Values in parentheses are given in nm.

^b^Only contributions above 10% are shown. H and L represent HOMO and LUMO, respectively.

**Figure 5:**
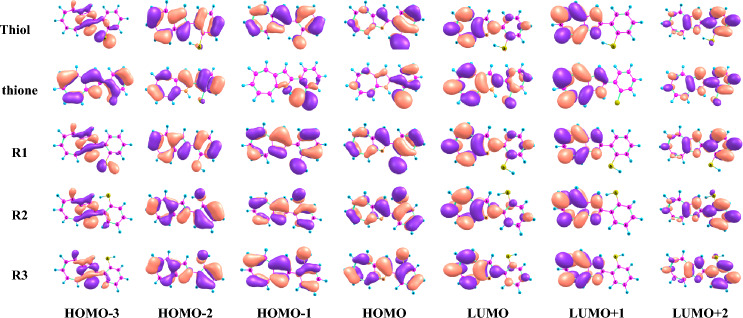
Frontier orbitals of the studied structures at the B3LYP/6-31G(d,p).

**Figure 6:**
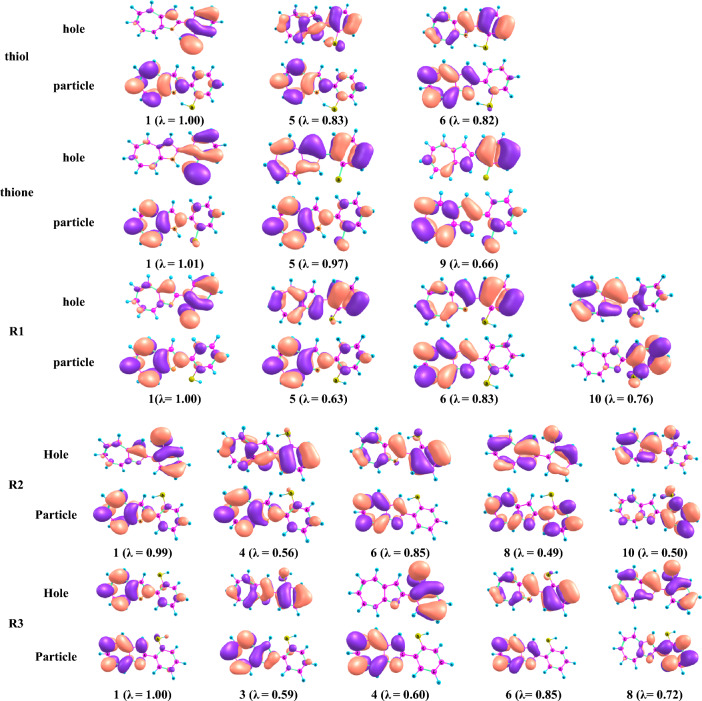
Natural transition orbitals (NTOs) for the excitation with significant and small but non-negligible oscillation strengths for the studied structures at the PBE/6-311+G(d,p) level with solvent effects of acetonitrile through SMD. The displayed occupied (holes) and unoccupied (electrons) NTO pairs are the only that have contributed more than 50% to each excited state (λ is eigenvalues of the pairs).

The strong electronic absorption of thione is attributed to the HOMO-3 to LUMO and HOMO-2 to LUMO + 1 transitions. The transitions from to HOMO-2 to LUMO and LUMO + 1 and HOMO-1 to LUMO + 1 cause maximum absorption peak for thiol, which appears at a lower wavelength than thione. However, the strong electronic absorption of the studied rotamers has a different contribution, as shown in Table 8. The NTOs for the high-intensity excited states of the investigated systems are shown in Fig. 5 to analyze the nature of absorption. The occupied and unoccupied NTOs are referred to as “hole” and “particles” transition orbitals, respectively. The NTOs generally give a simpler description of the excited state than the canonical orbitals. As displayed in Fig. 6, where the canonical orbitals were used, the dominant transitions are π-π* for the excitations, with some contribution from n-π* excitation. This makes the analysis of excitations cumbersome. However, as depicted in Fig. 6, the hole NTOs contributing to the illustrated band in Fig. 6 and Table 8 of all studied structures are delocalized over the whole molecular skeleton, while the particle NTOs are mainly delocalized over either azaazulene or benzene rings. This suggests π-π* excitation.

### Nonlinear optical (NLO) analysis

It is known that heterocyclic compounds have enhanced charge transfer, which could lead to large nonlinear optical properties. In this section, we will test this information and provide the scientific community any basic information about the hyperpolarizability of these tautomer's and rotamers. Non-linear optical properties (NLO) are the ability of any compound to convert light [with an intense electric field (LASER)] of a longer wavelength into light of a shorter wavelength. One of the non-linear optical phenomena is the second harmonic generation (SHG), where intense light of a longer wavelength is converted to half of the incident value upon absorption by the non-linear optical material.

Most applications of single crystals of any nonlinear material are evident in the fields of semiconductors, infrared detectors, solid state lasers, photosensitive materials, and crystalline thin films for microelectronics^85–87^. The investigation of the relationship between the electronic structure and NLO parameters of the studied compounds is calculated theoretically using ωB97XD/6–311++G(2d,2p) and B3LYP/6–311++g(2d,2p) (Table 9 and S2, respectively). Total static dipole moment (µ), the mean polarizability, the anisotropy of the polarizability ∆, α the mean first-order hyperpolarizability (*β*), the hyper-Rayleigh scattering (*β*_*HRS*_) and the depolarization ratio (*DR*) of the studied compounds are listed in Table 9. In this study, *P*-nitro aniline (PNA), a standard prototype molecule used in NLO studies, was chosen as a reference as there were no experimental values for the NLO properties of the studied compounds. The values of, ***α*** in Table 9 show that the order of increasing ***α*** with respect to PNA is: R2, Thiol, and R1 are ~ 3.5 and 3 times higher than PNA, respectively, whereas rotamer R3, and Thione is ~ 4 times higher than the standard PNA at ωB97XD/6–311 + +G(2d,2p). The calculated first order hyperpolarizability *β* of *p*- nitroacetanilide (PNA) is 15.5 × 10^–30^ esu as reported by T. Gnanasambandan et al.^88–90^ The analysis of the *β* parameter shows that Thiol, R2, R3, and R1 are ~ 2 times higher than PNA, while rotamer Thione is ~ 2.5 times higher than the reference at ωB97XD/6–311 + +G(2d,2p). Furthermore, for the investigated compound, the lowest value of *DR****,*** and the highest value of *β*_*HRS*_ confirm short bond length, indicating increased selectivity. Therefore, the studies tautomer's and rotamers show promising optical properties.

**Table 9:** Total static dipole moment (***μ***), the mean polarizability (<***α***>), the anisotropy of the polarizability (***Δα***), and the mean first-order hyperpolarizability (<***β***>), for the studied compounds (Thiol, Thione, R1, R2, and R3) computed at ωB97XD/6–311++G(2d,2p).

Property	PNA	Thiol	Thione	R1	R2	R3
*μ*_*x*_*,* D		− 4.1	− 8.0	− 2.1	3.2	1.7
*μ*_*y*_*,* D		− 2.5	− 4.2	− 1.6	0.4	0.9
*μ*_*z*_*,* D		0.0	0.0	0.2	− 0.6	− 0.5
*μ,* Debye^a^	2.4	4.8	9.0	2.7	3.3	2.0
*α*_*XX*_*,* a.u		− 81.0	− 83.2	− 77.2	− 81.2	− 75.2
*α*_*XY*_*,* a.u		− 5.7	− 11.0	− 0.1	4.6	− 0.7
*α*_*YY,*_ a.u		− 98	− 103.4	− 94.7	− 100.3	− 97.5
*α*_*ZZ*_*,* a.u		− 113.8	− 114.1	− 113.6	− 111.2	− 112.8
*α*_*YZ*_*,* a.u		1.3	0.0	− 0.4	− 1.2	− 1.1
*α*_*XZ*_*,* a.u		0.5	0.0	0.3	1.6	− 0.3
˂*α* > × 10^−24^ esu^b^	22	30.1	33.3	31.5	27.7	32.8
*Δα* × 10^−24^ esu		97.6	100.2	95.2	97.6	95.16
*βxxx,* a.u		− 58.9	− 102.7	− 32.9	56.6	28.8
*βxxy,* a.u		− 11.4	− 24.3	9.5	− 19.2	5.4
*βxyy,* a.u		− 27.5	− 52.5	− 5.1	17.1	0.1
*βyyy,* a.u		− 18.7	− 39.9	− 4.2	− 8.3	2.6
*βxxz,* a.u		− 0.7	0.0	2.2	− 4.9	6.2
*βxyz,* a.u		− 7.4	0.0	10.2	− 14.6	− 17.2
*βyyz,* a.u		0.9	0.0	2.5	− 3.9	− 1.9
*βxzz,* a.u		− 2.2	− 4.4	− 0.8	4.5	2.5
*βyzz,* a.u		1.8	1.1	2.8	3.9	1.8
*βzzz,* a.u		0.4	0.0	− 0.1	2.9	1.6
˂*β*˃ × 10^−30^ esu^c^	15.5	61.8	110.2	33.2	57.2	28.9
*DR*		0.3	0.0	2.8 × 10^−3^	0.4	0.1
*β*_*HRS*_		0.7	6 × 10^−4^	2.2	5.7	6.4

^a,b, c^PNA results are taken from ref^88–90^.

## Conclusions

Five tautomers and rotamers (thiol, thione, R1, R2, and R3) of 2-(2-Mercaptophenyl)-1-azaazulene were studied using density functional theory (DFT). At the B3LYP/6-31G(d,p), the geometrical structural parameters and vibrational frequencies have been discussed. According to the results, B97XD/6–311++G(2d,2p) performs better than CCSD(T)/6–311++G(2d,2p). A very good agreement is seen when compared to the similar molecule 2-(2-hydroxyphenyl)-1-azaazulene. All levels of calculations show that the thiol form is the most stable tautomer in the gas phase. The order of stability is reversed in ethanol as a polar solvent, whereas thione becomes the most stable tautomer. It can be seen that the thione NH1 proton and the thiol SH1 proton have high chemical shifts at $\delta$ 18.4 and 13.6 ppm, respectively. HOMA and NICS results for aromaticity provide a good match with the relative energies and the structure of the thiol-thione tautomer in both gas and ethanol solvent. The UV–vis absorption spectra of the studied compound have been calculated using the TD-PBE method with a 6–311+G(d,p) basis set for 2-(2-Mercaptophenyl)-1-azaazulene. UV–vis computations indicate that the thione form has a significant red-shift (extended to 1000 nm), followed by the thiol form (extended to 700 nm), and that the rotamers have the lowest red-shift (extended to 600 nm). NTOs are used to indicate the π − π* nature of the transitions. In comparison to the PNA molecule, the studied molecules offer good benefits in technology-related applications. According to the NLO study, tautomers and rotamers show promising optical properties.

### Supplementary Information

Supplementary Information.

## Data Availability

All data used or analysed during the current study are included in this published article [and its supplementary information files.
