# Supplementary material for: A density functional theory study of the molecular structure, reactivity, and spectroscopic properties of 2-(2-mercaptophenyl)-1-azaazulene tautomers and rotamers
Source: Sci Rep. 2023 Sep 20;13:15626. doi: 10.1038/s41598-023-42450-1 (PMC10511447; doi:10.1038/s41598-023-42450-1)
Supplement: Supplementary file 1 — Supplementary Information. [file 41598_2023_42450_MOESM1_ESM.docx]

**A density functional theory study of the molecular structure, reactivity, and spectroscopic properties of 2-(2-Mercaptophenyl)-1-azaazulene tautomers and rotamers**

Safinaz H. El-Demerdash^a^[[1]](#footnote-1)^*^, Shimaa Abdel Halim^b^, Ahmed M. El-Nahas^a^, Asmaa B. El-Meligy^a^[[2]](#footnote-2)^*^

^a^ Chemistry Department, Faculty of Science, Menoufia University, Shebin El-Kom 32512, Egypt

^b^ Chemistry Department, Faculty of Education, Ain Shams University, Cairo, Egypt

*

Table S1: The Cartesian coordinates of all optimized structures of the 2-(2-Mercaptophenyl)-1-azaazulene tautomer and rotamers with their corresponding transition states at B3LYP/6-31G d,p) in gas phase.

| 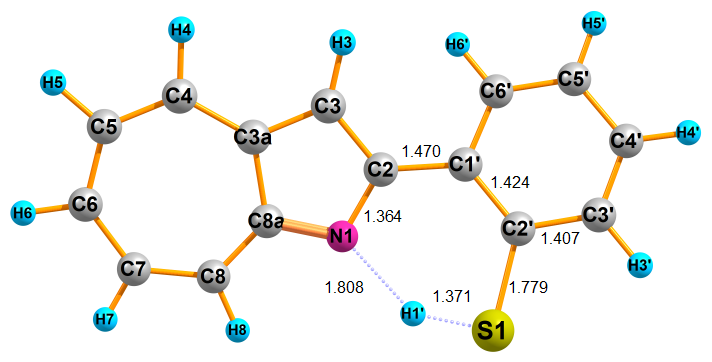  **Thiol** | 6 -4.854042000 0.164041000 0.011139000  6 -4.426788000 -1.161309000 -0.176335000  6 -3.133825000 -1.665380000 -0.222018000  6 -4.101271000 1.321158000 0.197737000  6 -1.922455000 -0.969494000 -0.093488000  6 -2.710678000 1.470671000 0.246016000  6 -1.737138000 0.484985000 0.118871000  1 -5.932502000 0.305765000 0.010369000  1 -5.221167000 -1.893169000 -0.301193000  1 -3.044434000 -2.739296000 -0.376648000  1 -4.672599000 2.237679000 0.322398000  1 -2.324654000 2.475015000 0.401591000  6 -0.629581000 -1.487896000 -0.143095000  1 -0.375477000 -2.523786000 -0.314295000  6 0.261864000 -0.398884000 0.030928000  7 -0.420224000 0.771841000 0.184396000  6 3.679821000 -1.964610000 0.175548000  6 2.310008000 -1.759877000 0.173206000  6 4.532756000 -0.863321000 0.052670000  1 1.655006000 -2.615998000 0.289257000  1 5.610450000 -0.998150000 0.050979000  6 1.729468000 -0.476302000 0.050614000  6 3.998902000 0.412228000 -0.055675000  1 4.661368000 1.268773000 -0.138782000  6 2.610395000 0.638030000 -0.053395000  16 2.129071000 2.343027000 -0.211104000  1 4.080720000 -2.968058000 0.277151000  1 0.795110000 2.095663000 -0.010277000 |
| --- | --- |
| 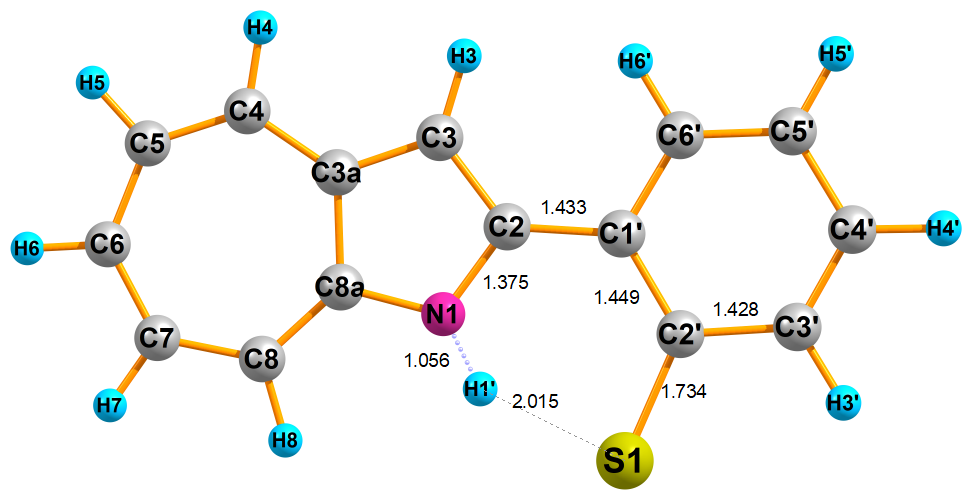  **Thione** | 6 4.840074000 0.175257000 -0.000013000  6 4.410443000 -1.174351000 -0.000083000  6 3.130799000 -1.686028000 -0.000085000  6 4.083143000 1.334182000 0.000042000  6 1.899861000 -0.984318000 -0.000010000  6 2.683376000 1.483491000 0.000049000  6 1.737122000 0.474262000 0.000041000  1 5.917797000 0.316970000 0.000006000  1 5.209551000 -1.911654000 -0.000124000  1 3.043645000 -2.770406000 -0.000130000  1 4.643741000 2.265500000 0.000082000  1 2.297964000 2.499763000 0.000059000  6 0.619775000 -1.526611000 0.000005000  1 0.390795000 -2.581231000 -0.000033000  6 -0.322930000 -0.464342000 0.000061000  7 0.398118000 0.706259000 0.000020000  6 -3.744615000 -1.921503000 0.000040000  6 -2.379962000 -1.773404000 0.000104000  6 -4.558409000 -0.765202000 -0.000077000  1 -1.754573000 -2.660103000 0.000175000  1 -5.640474000 -0.866271000 -0.000154000  6 -1.755326000 -0.490985000 0.000077000  6 -3.992541000 0.488421000 -0.000095000  1 -4.620391000 1.372985000 -0.000187000  6 -2.580554000 0.700011000 0.000029000  16 -2.022584000 2.342264000 -0.000021000  1 -4.190903000 -2.910705000 0.000069000  1 -0.144175000 1.611832000 -0.000063000 |
| 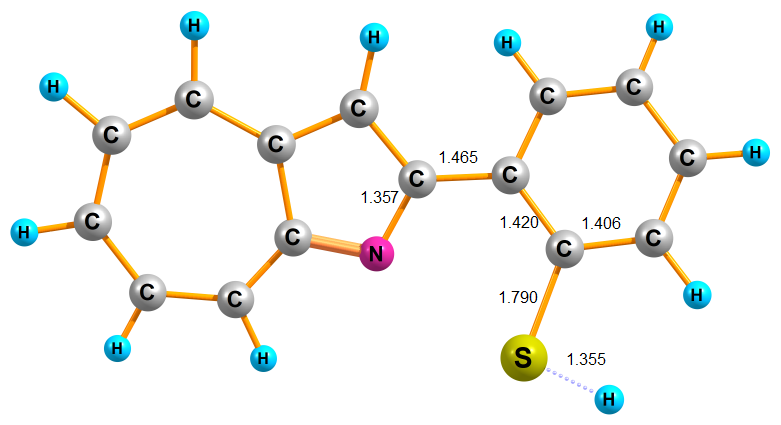  **R1** | 6 -4.822781000 0.193580000 0.023751000  6 -4.422878000 -1.138900000 -0.168971000  6 -3.139377000 -1.668616000 -0.221322000  6 -4.047662000 1.336653000 0.210493000  6 -1.915354000 -0.997422000 -0.094736000  6 -2.654686000 1.459453000 0.254104000  6 -1.699013000 0.456632000 0.121174000  1 -5.898509000 0.355934000 0.027363000  1 -5.232118000 -1.854549000 -0.293078000  1 -3.072654000 -2.743740000 -0.380258000  1 -4.601899000 2.263195000 0.338987000  1 -2.244842000 2.454298000 0.409129000  6 -0.630891000 -1.538470000 -0.149713000  1 -0.389120000 -2.578035000 -0.320632000  6 0.268747000 -0.456948000 0.023773000  7 -0.379007000 0.724185000 0.183536000  6 3.736194000 -1.918788000 0.169439000  6 2.357394000 -1.774298000 0.152337000  6 4.537889000 -0.779139000 0.074786000  1 1.732318000 -2.655946000 0.243850000  1 5.620586000 -0.863765000 0.087012000  6 1.732062000 -0.515540000 0.040863000  6 3.950825000 0.476047000 -0.029532000  1 4.588132000 1.352609000 -0.095483000  6 2.554846000 0.638866000 -0.049501000  16 1.877984000 2.288586000 -0.204308000  1 4.181915000 -2.904155000 0.260636000  1 3.089599000 2.848828000 -0.435013000 |
| 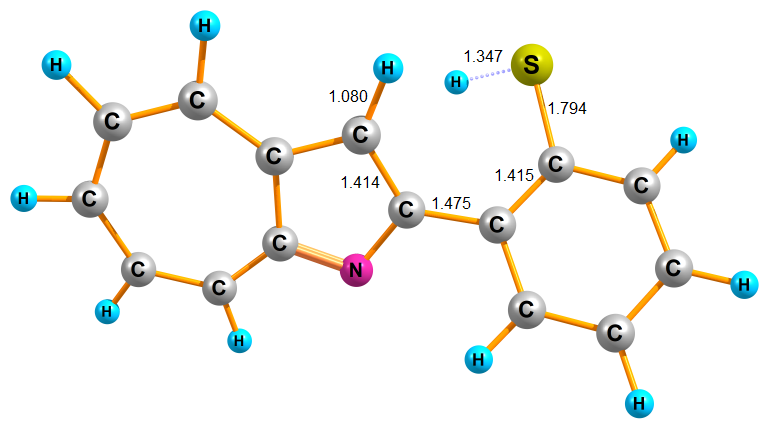  **R2** | 6 4.907594000 -0.026620000 0.004738000  6 4.291630000 1.115355000 -0.536066000  6 2.940193000 1.390844000 -0.691229000  6 4.331830000 -1.188665000 0.515472000  6 1.841284000 0.585441000 -0.344961000  6 2.977992000 -1.524573000 0.618688000  6 1.894680000 -0.745690000 0.238214000  1 5.993814000 0.000103000 0.025019000  1 4.969952000 1.893068000 -0.875528000  1 2.691892000 2.352786000 -1.133088000  1 5.026220000 -1.941296000 0.877350000  1 2.751625000 -2.502128000 1.038890000  6 0.477143000 0.905234000 -0.512792000  1 0.084763000 1.810826000 -0.949054000  6 -0.286322000 -0.172523000 -0.072550000  7 0.593367000 -1.146474000 0.368428000  6 -3.508847000 -2.050601000 -0.469287000  6 -2.160981000 -1.737834000 -0.369733000  6 -4.460205000 -1.047745000 -0.270317000  1 -1.426088000 -2.510250000 -0.577618000  1 -5.518720000 -1.275773000 -0.341609000  6 -1.725382000 -0.423942000 -0.086225000  6 -4.056372000 0.247698000 0.041401000  1 -4.800664000 1.012658000 0.236079000  6 -2.700152000 0.582247000 0.139269000  16 -2.358040000 2.294439000 0.531063000  1 -3.815256000 -3.062602000 -0.710280000  1 -1.267212000 2.092984000 1.296820000  1 0.300251000 -2.007826000 0.809281000 |
| 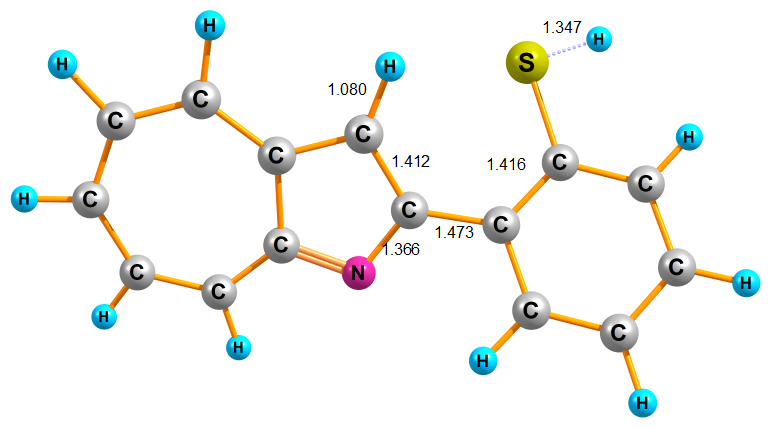  **R3** | 6 4.898188000 -0.032415000 -0.051196000  6 4.291113000 1.116081000 0.484308000  6 2.942117000 1.391210000 0.663394000  6 4.314214000 -1.203194000 -0.532848000  6 1.838697000 0.579056000 0.351799000  6 2.959756000 -1.542866000 -0.603586000  6 1.882350000 -0.760225000 -0.212996000  1 5.983700000 -0.004188000 -0.094322000  1 4.974326000 1.900705000 0.797008000  1 2.700696000 2.359860000 1.094133000  1 5.002711000 -1.959689000 -0.897951000  1 2.726729000 -2.526532000 -1.005433000  6 0.477261000 0.898418000 0.544028000  1 0.098285000 1.809484000 0.980751000  6 -0.292460000 -0.183229000 0.127179000  7 0.580075000 -1.164189000 -0.313083000  6 -4.048322000 0.272829000 -0.089838000  6 -2.685149000 0.599932000 -0.124163000  6 -4.472360000 -1.022439000 0.186204000  1 -5.535228000 -1.240630000 0.207772000  6 -1.732133000 -0.421375000 0.131275000  6 -3.541245000 -2.034310000 0.436620000  1 -3.868715000 -3.042132000 0.666200000  6 -2.189792000 -1.729474000 0.404784000  1 -4.788306000 1.041042000 -0.288856000  1 -1.467829000 -2.504016000 0.647463000  16 -2.163583000 2.251631000 -0.545492000  1 -3.401784000 2.746057000 -0.727183000  1 0.278798000 -2.024730000 -0.749909000 |
| 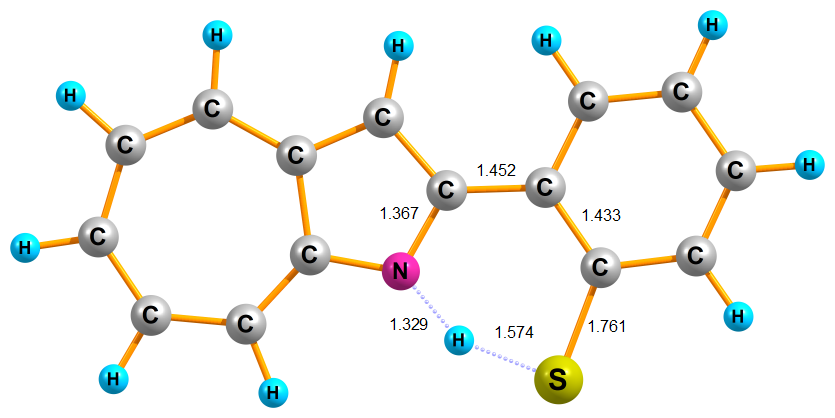  **TST** | 6 4.820049000 0.173523000 0.000019000  6 4.405316000 -1.173311000 0.000103000  6 3.122781000 -1.694267000 0.000114000  6 4.053950000 1.332648000 -0.000090000  6 1.896023000 -1.002387000 0.000047000  6 2.658283000 1.471930000 -0.000143000  6 1.706253000 0.462324000 -0.000078000  1 5.896824000 0.325122000 0.000039000  1 5.209048000 -1.905527000 0.000168000  1 3.045394000 -2.779805000 0.000184000  1 4.611676000 2.265728000 -0.000145000  1 2.264119000 2.484902000 -0.000242000  6 0.614141000 -1.547614000 0.000068000  1 0.381472000 -2.602135000 0.000175000  6 -0.307795000 -0.471484000 -0.000036000  7 0.376211000 0.712439000 -0.000129000  6 -3.775907000 -1.890101000 -0.000091000  6 -2.400810000 -1.766598000 -0.000110000  6 -4.564468000 -0.727212000 0.000003000  1 -1.791175000 -2.664280000 -0.000199000  1 -5.648215000 -0.805191000 0.000021000  6 -1.759263000 -0.503390000 -0.000038000  6 -3.969418000 0.521633000 0.000064000  1 -4.582488000 1.417239000 0.000126000  6 -2.564941000 0.682211000 0.000039000  16 -1.932145000 2.325379000 0.000108000  1 -4.238372000 -2.871872000 -0.000153000  1 -0.472605000 1.735250000 -0.000051000 |
| 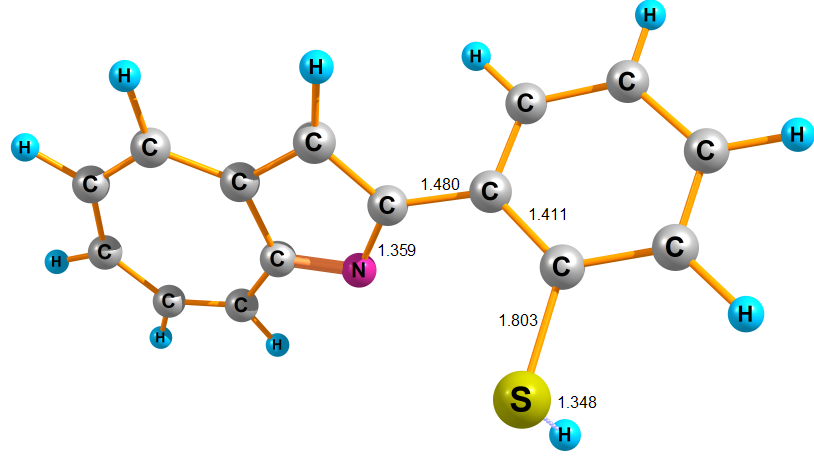  **TSR1** | 6 4.844735000 0.135180000 -0.119762000  6 4.401456000 -0.973130000 0.613572000  6 3.097492000 -1.407771000 0.841710000  6 4.109377000 1.098916000 -0.813277000  6 1.903127000 -0.837568000 0.394724000  6 2.725023000 1.213695000 -0.955619000  6 1.731700000 0.385605000 -0.435380000  1 5.924629000 0.263636000 -0.153763000  1 5.184137000 -1.578371000 1.064602000  1 2.994460000 -2.308708000 1.444622000  1 4.696428000 1.868237000 -1.308937000  1 2.348587000 2.049952000 -1.539616000  6 0.595194000 -1.280001000 0.625440000  1 0.294007000 -2.138771000 1.210406000  6 -0.253771000 -0.358589000 -0.021589000  7 0.425939000 0.630778000 -0.659092000  6 -3.644968000 -1.890654000 -0.600068000  6 -2.274596000 -1.709907000 -0.447999000  6 -4.509559000 -0.818727000 -0.374938000  1 -1.594520000 -2.532999000 -0.644827000  1 -5.582563000 -0.943211000 -0.487282000  6 -1.728151000 -0.463101000 -0.089361000  6 -3.993675000 0.411749000 0.023720000  1 -4.666367000 1.233165000 0.249267000  6 -2.613324000 0.606618000 0.163537000  16 -2.020473000 2.181155000 0.812222000  1 -4.035168000 -2.859781000 -0.895923000  1 -2.557995000 2.959028000 -0.148705000 |
| 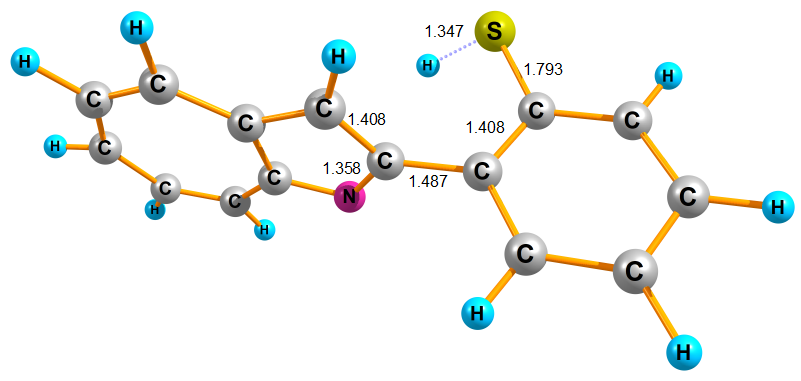  **TSR2** | 6 -4.839565000 -0.031456000 -0.107317000  6 -4.309951000 0.297753000 1.149154000  6 -2.979797000 0.325132000 1.557555000  6 -4.186855000 -0.417685000 -1.277676000  6 -1.836285000 0.028318000 0.809395000  6 -2.816725000 -0.580800000 -1.506839000  6 -1.766173000 -0.393792000 -0.614546000  1 -5.923872000 0.023453000 -0.178383000  1 -5.040601000 0.569620000 1.906995000  1 -2.804262000 0.615830000 2.592070000  1 -4.831419000 -0.620681000 -2.129289000  1 -2.509410000 -0.893659000 -2.501845000  6 -0.500597000 0.058294000 1.227353000  1 -0.128783000 0.323465000 2.207696000  6 0.269177000 -0.325281000 0.112193000  7 -0.480690000 -0.593708000 -0.988140000  6 3.694295000 -1.920292000 0.293068000  6 2.313373000 -1.738275000 0.266640000  6 4.535760000 -0.821246000 0.119450000  1 1.648559000 -2.586222000 0.400524000  1 5.614289000 -0.948126000 0.134228000  6 1.749155000 -0.468531000 0.082401000  6 3.995073000 0.443672000 -0.097976000  1 4.649397000 1.293157000 -0.268861000  6 2.606872000 0.630317000 -0.118567000  16 2.011635000 2.300431000 -0.382641000  1 4.108651000 -2.912016000 0.445962000  1 0.829577000 1.967491000 -0.935603000 |
| 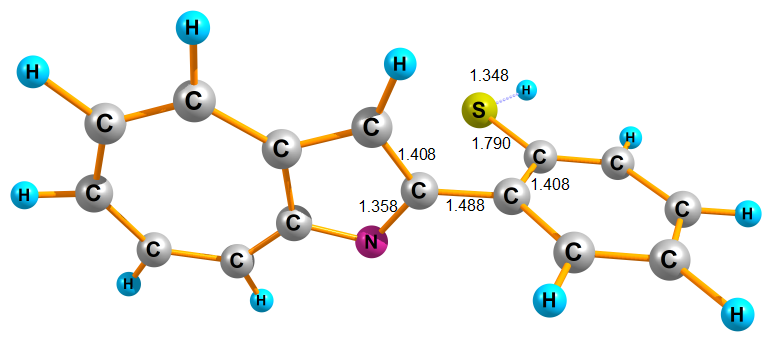  **TSR3** | 6 -4.830965000 0.006113000 -0.139476000  6 -4.324479000 -0.051672000 1.166328000  6 -3.000844000 -0.152055000 1.587687000  6 -4.157108000 -0.020992000 -1.361103000  6 -1.845072000 -0.220310000 0.805634000  6 -2.783850000 -0.114164000 -1.604487000  6 -1.748486000 -0.202064000 -0.678706000  1 -5.913948000 0.084246000 -0.210003000  1 -5.068490000 -0.011854000 1.958189000  1 -2.844544000 -0.180490000 2.664935000  1 -4.786755000 0.039073000 -2.245360000  1 -2.457941000 -0.118949000 -2.641804000  6 -0.515968000 -0.322703000 1.235756000  1 -0.161338000 -0.359891000 2.256887000  6 0.271918000 -0.357355000 0.069566000  7 -0.458883000 -0.287358000 -1.072950000  6 3.717128000 -1.923366000 -0.053772000  6 2.334288000 -1.757804000 -0.009180000  6 4.540357000 -0.797850000 -0.072273000  1 1.680737000 -2.624870000 0.002423000  1 5.620325000 -0.908180000 -0.107258000  6 1.753814000 -0.483759000 0.018945000  6 3.983200000 0.478697000 -0.048895000  1 4.633266000 1.348526000 -0.069545000  6 2.593034000 0.646933000 -0.002383000  16 1.840371000 2.271313000 0.023901000  1 4.146252000 -2.920202000 -0.075092000  1 2.996881000 2.957201000 0.113030000 |

Table S2: Total static dipole moment (***μ***), the mean polarizability (***˂α˃***), the anisotropy of the polarizability (***Δα***), and the mean first-order hyperpolarizability (***˂β˃***), for the studied compounds (**Thiol, Thione, R1, R2, and R3**) computed at B3LYP/6-311++g(2d,2p).

| **Property** | **PNA** | **Thiol** | **Thione** | **R1** | **R2** | **R3** |
| --- | --- | --- | --- | --- | --- | --- |
| ***μ_x_,* D** |  | -6.25 | 13.40 | -2.95 | -4.92 | -2.67 |
| ***μ_y_,* D** |  | -4.0 | -6.82 | -2.82 | 1.11 | 1.79 |
| ***μ_z_,* D** |  | -0.02 | 0.00 | 0.65 | 1.37 | 1.07 |
| ***μ,* Debye ^a^** | 2.44 | 7.42 | 15.04 | 4.13 | 5.22 | 3.39 |
| ***α_XX_,* a.u.** |  | -72.78 | -77.00 | -66.02 | -71.25 | -62.64 |
| ***α_XY_,* a.u.** |  | -7.62 | 16.83 | 0.26 | -6.09 | 0.66 |
| ***α_YY,_* a.u.** |  | -97.27 | -105.17 | -93.62 | -100.92 | -97.72 |
| ***α_ZZ_,* a.u.** |  | -114.94 | -115.48 | -114.12 | -111.84 | -112.67 |
| ***α_YZ_,* a.u.** |  | 1.233 | 0.0 | -0.66 | 2.07 | 1.19 |
| ***α_XZ_,* a.u.** |  | 0.59 | 0.00 | 0.54 | 1.91 | -0.80 |
| **˂*α*> ×10^−24^ esu^b^** | 22 | 39.04 | 45.17 | 41.83 | 38.19 | 44.56 |
| ***Δα* ×10^−24^ esu** |  | 95.00 | 99.22 | 91.25 | 94.67 | 91.01 |
| ***βxxx,* a.u.** |  | -97.21 | 191.46 | -54.10 | -89.012 | -52.83 |
| ***βxxy,* a.u.** |  | -20.32 | -44.12 | 7.74 | -20.32 | 7.97 |
| ***βxyy,* a.u.** |  | -36.53 | 76.55 | -6.07 | -20.33 | -0.40 |
| ***βyyy,* a.u.** |  | -33.84 | -67.91 | -13.92 | -6.89 | 5.29 |
| ***βxxz,* a.u.** |  | -0.37 | 0.00 | 4.78 | 4.22 | -8.44 |
| ***βxyz,* a.u.** |  | -6.56 | 0.00 | 18.16 | -20.42 | -25.16 |
| ***βyyz,* a.u.** |  | 0.97 | 0.00 | 4.44 | 7.67 | 3.75 |
| ***βxzz,* a.u.** |  | -4.37 | 10.55 | -2.77 | -10.72 | -5.49 |
| ***βyzz,* a.u.** |  | 0.51 | -2.21 | 1.57 | 3.60 | 3.59 |
| ***βzzz,* a.u.** |  | 0.39 | 0.0007 | 0.45 | -0.19 | -0.65 |
| **˂*β*˃ × 10^−30^ esu^c^** | 15.5 | 102.93 | 203.15 | 55.86 | 89.28 | 53.10 |
| ***DR*** |  | 1.14 | 1.96 | 8.80×10^−3^ | 2.08×10^−3^ | 5.95×10^−3^ |
| ***β_HRS_*** |  | 0.54 | 8.60×10^−4^ | 4.80 | 4.23 | 8.47 |

1. * Corresponding authors:

   [asmaaphys@yahoo.com](mailto:asmaaphys@yahoo.com) (Asmaa B. El-Meligy ) [↑](#footnote-ref-1)
2. [hamdysafinaz@yahoo.com](mailto:hamdysafinaz@yahoo.com) (Safinaz H. El-Demerdash) [↑](#footnote-ref-2)
